# Developing Interpersonal Trust Between Service Users and Professionals in Integrated Services: Compensating for Latent Distrust, Vulnerabilities and Uncertainty Shaped by Organisational Context

**DOI:** 10.5334/ijic.5599

**Published:** 2021-07-01

**Authors:** Rie Mandrup Poulsen, Kathrine Hoffmann Pii, Lene Falgaard Eplov, Mathias Meijer, Ute Bültmann, Ulla Christensen

**Affiliations:** 1 The National Board of Social Services, Center for Data, Analysis and Methods, Edisonsvej 1, 5000 Odense C, Denmark; 2 University College Copenhagen, Faculty of Health, Institute for Nursing and Nutrition, Tagensvej 86, 2200 Copenhagen N, Denmark; 3 Copenhagen Research Center for Mental Health – CORE, Mental Health Center Copenhagen, Copenhagen University Hospital, Kildegårdsvej 28, Entr. 3A, 4th floor, 2900 Hellerup, Denmark; 4 University of Groningen, University Medical Center Groningen, Department of Health Sciences, Community and Occupational Medicine, Hanzeplein 1, FA 10, 9700 RB Groningen, The Netherlands; 5 Department of Public Health, Section of Social Medicine, University of Copenhagen, Øster Farimagsgade 5, P.O.B. 2099, 1014 Copenhagen K, Denmark

**Keywords:** trust, integrated services, common mental disorders, organisational context, person-centred services, process evaluation

## Abstract

**Introduction:**

Studies show a need for trust between stakeholders in integrated services. However, few studies have investigated how trust develops between stakeholders on a micro-level. In a Danish intersectoral intervention for persons on sick leave due to common mental disorders, we explored why trust is needed and how trust is developed between micro-level stakeholders.

**Methodology:**

The qualitative study was based on 12 observations of inter-organisational meetings, 16 interviews with service users, 24 interviews with health care professionals and employment consultants, and 8 interviews with supervisors. The analysis was guided by the theoretical concepts (dis-) trust, vulnerability and uncertainty.

**Results:**

Latent distrust between involved organisations, and vulnerabilities and uncertainties related to employment consultants’ statutory power over service users caused a perceived need for interpersonal trust. Time to establish knowledge-based relationships, healthcare professionals’ caring approach, and creating a feeling of sharing interests were compensating trust-building strategies that were often regarded as positive.

**Discussion and conclusion:**

Trust in personal relationships between stakeholders appeared to compensate for contextually shaped distrust, vulnerability and uncertainty. Identifying latent distrust, vulnerabilities, uncertainties, and power structures might be key to improving trust-building strategies in a specific context. The time-consuming process of trust-building between micro-level stakeholders should be supported structurally.

## Introduction

Trust among stakeholders is a central issue in integrated care and integrated services (e.g., between health care and social services) [1,2,3]. As described in the Rainbow model for integrated care by Valentijn et al.] [4] and supported by other research [2,5,6], trust is important for the integration of services at all societal levels: between service users and professionals (micro-level), between professionals and managers (meso-level), and between policy makers (macro-level) from involved organisations and systems. The need for trust in integrated services is studied widely on the organisational meso-level where it is hypothesised as being important for different reasons. First, groups of professionals and managers often collaborate without formalised inter-organisational hierarchies, which can create uncertainty about decisional mandates [7,8]. Accordingly, control-loss due to unclear mandates and new decisional structures is suggested to increase the need for trust [6,9]. Second, integration of services increases the risk of having institutional norms and territory control questioned by the collaborating organisation [10]. Third, integration of services can blur the lines between professional territories and create doubt about new roles and responsibilities [11]. Trust in collaborators is suggested to counteract exploitation whilst new roles are negotiated [12]. Therefore, trust has been described as ‘the glue that makes partnerships work’ [1] because trust counteracts the risk of opportunistic actions for intra-organisational gains (e.g., economic profit, credit for shared tasks, or reputation) [9,13,14].

Although trust is supposed to be critical for the success of inter-organisational and integrated services [8], it is rarely naturally present [11]. Most trust theories take a developmental perspective on trust [6]. Trust is theorised as being developed over time through reinforcing interaction and nurturing cycles whereby stakeholders manage diverging interests, power imbalances, and vulnerabilities [9], which might vary between stakeholders who enter the collaboration [6]. For instance, less powerful stakeholders are expected to need more trust in collaborators compared with powerful stakeholders [9,15].

The development of trust between organisations has been thoroughly investigated [6,9,12,16]. However, it is recommended that more studies investigate trust-building between micro-level stakeholders in integrated service delivery [17]. Although distrust between professionals is a common problem in integrated services [18], there is little knowledge about how this might affect service users and how the problem of distrust can be resolved. A few previous studies suggested that users of integrated services experience the same trust-related needs as do those of non-integrated services, e.g., a need to trust that professionals understand their needs [19], will act to support those needs [20], and are genuinely interested in caring for them [21]. However, the development of trust between service users and more than one professional in integrated services has, to the best of our knowledge, not been investigated.

### Aim

This study describes why personal trust is needed and how the development of trust is supported between three micro-level stakeholders: service users, care managers from mental health care centres and employment consultants from jobcentres. Firstly, the perceived need for trust is described through identification of latent distrust and key vulnerabilities and uncertainties among the three stakeholders. Secondly, we describe compensating strategies applied or experienced by the three stakeholders in the development of trust.

### Study context: the Danish IBBIS intervention

The empirical setting for this study was a newly developed, person-centred, intersectoral integrated intervention named IBBIS. This study is part of a process evaluation supplementing an effectiveness evaluation of the IBBIS intervention delivered from April 2016 to December 2018 (see study protocols [22,23] for more information). The IBBIS intervention was designed to integrate therapeutic treatment in mental health care (public health care sector) with vocational rehabilitation services in Danish municipal jobcentres (public sector organisation administrating the comprehensive social security [24]). This integration aimed to improve return-to-work outcomes for the target group: persons on sick leave due to common mental disorders (depression, anxiety and stress). This target group often face problems with returning to work because of cognitive impairments [25], feelings of loss of control over their situation (e.g. economy) [26], fear of stigma or distrust in the validity of their illness (e.g. because stress is not unambiguously perceived as an objectively validated illness) [27,28], possible disputes about their diagnosis [29], and professionals’ difficulties in determining future work capabilities [26,28,30].

Integration of the services of the care manager and the employment consultant was designed to accommodate a need for better coherence of services [30,31,32,33,34]. Designed on the basis of the theory of relational coordination by Jody Hoffer Gittell [35], the integration included mandatory inter-organisational roundtable meetings, inter-organisational supervision and co-location of professionals in municipal offices [36]. The roundtable meetings were the central forum for knowledge-sharing between service users, care managers, and employment consultants; the creation of shared goals with the service users; and a shared plan for treatment and return to work. This multidisciplinary assessment was considered to initiate the integrated intervention course shortly after monodisciplinary assessments by both professionals. Furthermore, medical notes for the sick leave case were produced by healthcare professionals from the integrated team. Despite the comprehensive structural integration efforts, the first process evaluation study showed that the collaboration initially led to conflicts, competition, and scepticism between professionals [37].

Service users were referred to the IBBIS intervention by a jobcentre and received sickness benefit from the jobcentre. The employment consultants practised both support and control regarding the service users. The latter entailed continuous assessment of service users and decisional authority to determine eligibility for sickness benefit and other benefits based on their health, workability and compliance to sick leave legislation [38]. The health care professionals were solely committed to supporting the service users through talk therapy (cognitive behavioural therapy or coaching) and could not use coercion towards the service users. The IBBIS intervention was launched and tested as part of a large reform of the Danish sickness benefit legislation which aimed to fasten provision of support and thereby return-to-work rates [39]. The primary amendments of the reform involved the default length of sickness benefit being shortened from 52 weeks to 22 weeks, and thus, week 22 being given status as the ‘new reassessment time’. Prolongation of sickness benefit beyond week 22 was possible cf. seven new legal paragraphs for eligibility (some required medical evidence from the health care sector) [40]. A new programme was introduced to support service users who were not eligible for sickness benefit after week 22 with a lower benefit level, the ‘assessment programme’ [39].

## Methods

This study focused on trust in the service provision (micro-level) of an intersectoral integrated intervention [41], i.e., the interactions between professionals and service users, but took a contextual focus on the organisational, legislative and social factors that influenced micro-level service provision in the intervention, as recommended by Craig et al. [42].

### Empirical material

This study was conducted through secondary analysis of empirical material (12 observations and 46 interviews with service users, professionals and supervisors) from two process evaluation studies about the roundtable meetings [37,43], and primary analysis of two additional interviews with supervisors that were conducted specifically for this study. The large empirical material ensured sufficient information power from interviews with sparse ‘specificity’ regarding the perceptions and practices regarding trust [44], e.g., expressions and experiences regarding trust and distrust were sparsely covered in each interview. Secondary analysis of the empirical material was relevant because stakeholder-trust emerged as an important theme in the original studies and was not sufficiently investigated and disseminated in the primary analysis [45]. See overview of empirical material for this study in ***Table 1***.

**Table 1:** Overview of empirical material.


DATA TYPE	PARTICIPANTS	N=	SERVICE USER CHARACTERISTICS	RESEARCHER	PERIOD	AVERAGE LENGTH: MINUTES (MIN–MAX)	PRIMARY/SECONDARY ANALYSIS

Observations of RTM	SU, CM, and EC	12	8 with stress, 1 with anxiety 3 with depression	RMP	April 2017– January 2018		Secondary
	
Interviews early after RTM, face-to-face	CM	12				55 (27–93)	Secondary
	
	EC	12					Secondary

Interviews early after RTM, telephone	SU	10	8 with stress 1 with anxiety 1 with depression	KHP	January 2017–March 2018	25 (13–34)	Secondary
		
Follow-up interviews, face-to-face		6				55 (35–72)	Secondary

Interviews, face-to-face	Supervisors	8	–	RMP	August 2017–June 2019	61 (54–79)	Secondary (6) and primary (2)


RTM: roundtable meeting; SU: service user; CM: care manager; EC employment consultant; RMP: Rie Mandrup Poulsen; KHP: Kathrine Hoffmann Pii.

To investigate the practices of the service users, care managers, and employment consultants, RMP observed 12 roundtable meetings with 12 service users. No roundtable meetings were observed by more than one researcher, but KHP observed 20 other roundtable meetings, and observations were discussed continuously to enhance reflectivity and validity. Further, RMP conducted 24 semi-structured face-to-face interviews with the care managers and employment consultants who were present during the 12 observed roundtable meetings. Interviews with professionals focused on the roundtable meeting as a forum to create integration of services [37]. For the same process evaluation, KHP conducted telephone interviews with 10 other service users immediately after their first roundtable meeting. Service users were recruited for observation and interviews through the professionals. The interview guide addressed service users’ perception of the roundtable meeting with a focus on person-centred services and shared decision-making [43]. To describe the service users’ perception of the integrated intervention more generally, KHP conducted face-to-face follow-up interviews with six of these individuals (on average six months after their first roundtable meeting).

As recommended in process evaluations [42], the organisational context that influenced the inter-organisational meetings was also investigated: RMP conducted eight semi-structured interviews with supervisors and managers from the mental health care services and the jobcentre (described as supervisors to allow anonymity of a small group of managers). Most of the interviewed supervisors were directly involved in the integrated practice (e.g., had consultations with service users) and their perspective was included to gain insight into the relationship between micro-level practice and the organisational context. The two interviews that were conducted specifically for this study focused on supervisor’s experience of how trust and distrust affected the daily work in the integrated intervention.

Service users and professionals were invited with a purposive sampling strategy to investigate practices from four different locations and service users with different needs [37]; most of the involved professionals were interviewed [46]. The researchers involved in the interviews and observations had no previous relationship with service users, but RMP was familiar with most of the professionals through the IBBIS effectiveness trials. All professionals agreed to participate, and there was no registration of service users who did not participate. All interviews were audio-recorded and transcribed verbatim. No interviews were repeated, and neither transcripts nor findings were returned to participants for comments.

### Ethical considerations

All data were handled in compliance with the General Data Protection Regulation (GDPR EU REGULATION 2016/679) and the Data Protection Act (Act No. 502 of 23 May 2018, Denmark). The research project has been approved by the Regional Ethics Committees of the Capital Region of Denmark (# H-16015724). Participants were informed about the purpose and methods of the study and the principle of voluntariness. Informants were anonymised and were randomly denominated “he” or “she” to reduce the risk of recognition.

### Analytical framework and procedure

The 16 interviews conducted by KHP were received by RMP for this study as un-coded verbatim transcripts without the original audio files [47]. The whole interview material was first coded with Nvivo 12 software by RMP. The initial coding aspired an inductive coding strategy. To enhance the validity of the analysis, UC and RMP discussed coding and samples of the material and reached consensus through discussion.

The analysis for this study was performed in an abductive process that alternated between analytical readings of the empirical material, coding of the material, and reading of other empirical and theoretical literature [48]. The theoretical focus on trust took a point of departure in the Rainbow model for integrated care where trust is an important aspect of normative integration [3]. A theoretical analytical framework was developed after the empirical material was produced and focused on the stakeholders’ expressions and practices related to the four analytical concepts trust, distrust, vulnerability, and uncertainty. In this study, trust is defined as an interpersonal attitude needed when there is uncertainty about the actions of others, and when these actions pose a threat to the person who is vulnerable to these actions [6,49]. The concepts trust, distrust, vulnerability, and uncertainty are interrelated. Although trust is an enabling factor that encourages persons to accept vulnerability and engage in actions with uncertain outcomes [9,15], a high degree of uncertainty and vulnerability also makes trust more risky and creates incentive to distrust [49]. Interpersonal trust is theorised to be developed in reciprocal iterative trust-building cycles in the relationship between persons who present themselves as competent and willing to act in a way that will not exploit the other part’s vulnerability [9]. Trust is granted when the recipient feels safe and this presentation is perceived as trustworthy [51].

The analytical procedure was inspired by Graneheims’ content analysis [51] and was conducted by producing analytical tables (example in appendix 1). Quotes were analysed into stakeholder-specific condensed meaning units, stakeholder-specific themes and general (inter-stakeholder) themes to generate overviews of stakeholder perceptions. Field notes regarding roundtable meetings were analysed without stakeholder-specification. Analytical tables were produced through continuous discussion and reflection between the authors. Further, RMP and KHP discussed the secondary analysis of the service-user interviews and observations, as recommended by Hinds et al. [45]. Key quotes are presented in the findings in an attempt to balance the perceptions of the three types of stakeholders. Service-user quotes are marked as either ‘initial’ or ‘follow-up’ to indicate the time point.

## Findings

Firstly, we describe why distrust was latent and interpersonal trust appeared to be needed between the three types of stakeholder. The latter is done by describing service users’ vulnerability as well as service users’ and care managers’ perceived uncertainty related to the employment consultants’ use of power. Secondly, we describe some of the strategies that were applied (more or less deliberately) to develop interpersonal trust.

### Organisational context and distrust in systems

The organisational context of the integrated intervention appeared to shape the need for trust. In particular, two types of abstract perceptions of the involved organisations (also referred to as systems) indicated a predetermined scepticism and latent distrust from which the three stakeholders started their collaboration.

Firstly, based on previous experiences or predetermined perceptions, some professionals expressed a subtle distrust in the organisation they did not belong to themselves. This was not expressed as a manifest distrust in specific persons but rather as a distrust in abstract systems. For instance, professionals referred to a perception in the mental health care services that the jobcentre sometimes misused information from sickness certifications and a perception in the jobcentre that some medical doctors misused sickness certifications to prolong sickness benefit. A supervisor expressed this more directly:

“It is sort of an ingrown distrust between the systems that we build on.” (Supervisor, mental health care)

Secondly, some service users and care mangers expressed negative perceptions of, or experiences with, the abstract jobcentre system before entering the integrated intervention that influenced their expectations and interpersonal attitudes early in the collaboration. Some service users felt threatened by the jobcentre system and had little trust that it would support them. The following paragraphs elaborate how the jobcentre context shaped the need for trust in more concrete ways.

### Service-user vulnerability

Care managers vocalised that service users were vulnerable due to the combination of their mental state and the employment consultants’ statutory power over them. A care manager exemplified this by describing a service user with anxiety who referred to the jobcentre hallway as the “death row” and concluded that interaction with the jobcentre worsened her anxiety. Service users described or displayed their vulnerability less explicitly. Three of 12 service users cried during the observed roundtable meetings; others expressed fear that the employment consultants would force them to start a particular job. A service user described her vulnerability:

“She [employment consultant] said to me: “(distorted voice) Can you give me a single reason why I should not declare you fit for work?” That question does not open a dialogue with a vulnerable person! […] If IBBIS is supposed to have some sort of care-giving effect on a person who is vulnerable like I am, then you really need to change the rhetoric.” (Service user, follow-up)

Employment consultants generally vocalised service users’ vulnerability less in interviews. However, a supervisor described the double-vulnerability concretely:

“If you [service user] are sick and insecure and vulnerable, and you have experienced something at work, that was unhealthy for you, then, naturally, you would be afraid to do it again. […] Because they [service users] are very aware that the jobcentre has some power over them. In that situation, they can start to worry, how are they [employment consultants] going to use that power?” (Supervisor, Jobcentre)

### Uncertainty about employment consultants’ interests

Furthermore, service users and care managers sometimes felt uncertain about how the individual employment consultants would balance the interests of the service user and the jobcentre when exercising this power. Sometimes, interest appeared overlapping, e.g. in observations. However, at other times, stakeholders became aware of diverging interest of the service user and the jobcentre:

“The employment consultant handles the work-related and the legislative stuff. The latter is not that important for me. But it is very important for the jobcentre [laughing softly]. […] In the beginning, the employment consultant sort of pushed me… for a job assessment, or whatever the term was. And I did not feel quite ready for that.” (Service user, initial interview)

In cases of diverging interests, some care managers initially felt uncertainty regarding individual employment because they perceived employment consultants as handling the diverging interests very differently. This uncertainty could make care managers enter the integrated collaboration and knowledge-sharing with some caution:

“The employment consultants are so different from each other. Some of them are very liberal. For instance, if the service user cancels a meeting too late – then it is OK [the service user is not sanctioned]. […]. I think you must be careful. First you need to become familiar with your employment consultant. Because you can destroy things for the service user.” (Care manager)

Some employment consultants confirmed that employees of the jobcentre handled the balance between jobcentre and service-user interests differently:

“Within the limits of the legislation… I go very far. As far as I can and sometimes a little bit more. If that is what it takes for that individual. […] I have colleagues who close a case due to no-show.” (Employment consultant)

Even in cases where conflicts of interests were not explicit, a supervisor suggested that service users could feel uncertain about whether recommendations from the employment consultant were based on the interests of the jobcentre or the service user:

“The service user can feel very uncertain […]. Can I trust what the jobcentre says? What are the rationales for saying what they are saying to me? Is it because over there in the jobcentre they need to save money [referring to the reform]?” (Supervisor, jobcentre)

### Compensating strategies and trust building

In the context of the above-mentioned latent distrust, vulnerabilities, and uncertainties, the service users and professionals often developed interpersonal trust. We identified the following strategies which supported trust-building to some extent and describe how the professionals had different premises for trust-building.

#### Service users’ trust in professionals

Some service users described how trust in the individual professional was gradually developed over time and over several sessions and how it contributed to their engagement and knowledge-sharing regarding sensitive issues:

“It takes some sessions before you feel safe enough to tell things. It has been that way with [the employment consultant] and [the care manager]. There are many things I have not disclosed yet. But the further we go, and I know where I have got them, the more I dare to trust them with those problems that are actually there.” (Service user, follow-up interview)

Some service users explained how the feeling of sharing interests with professionals was important for their engagement in the programme:

“It was vital for me […] that I sort of knew them. So that I do not feel like it is them against me in any way. I mean, I can actually feel that we want the same thing.” (Service user, initial interview)

Some service users expressed that trust in the personal relations between them and the individual professionals was important for their willingness to engage actively in the roundtable meeting which could otherwise be perceived as a ‘two-against-one situation’.

#### Care manager strategy

Interviews and observations showed that care managers’ professional training, services and organizational dissociation with the jobcentre affected their possibilities for trust-building. Care managers used and vocalised caregiving strategies to establish service users’ trust in themselves and employment consultants. Care managers expressed that they compensated for the service users’ potential distrust or scepticism towards the jobcentre authority with a caring, non-authoritative approach and sometimes explicit distance towards the jobcentre.

Most service users were positively surprised by the approach towards them in the IBBIS team, and some service users explained how they became more comfortable because of the care manager’s approach:

“That [roundtable meting] was really difficult for me. […] But it went alright with the care manager’s guidance and with what she could apparently read between the lines. I think she did that very well. […] That showed me that they take care of me and that was very positive.” (Service user, initial interview)

Several observations showed that care managers also expressed care through supporting service users’ individual interests (e.g. the wish to postpone the planning of a concrete return-to-work date). The care manager’s primary task, therapy, was mentioned explicitly as supporting the development of trust in professionals:

“I think service users are positively surprised in IBBIS. […] They don’t just get a jobcentre authority, they are also offered care and a chance to get mentally well. Care is an aspect of therapy that creates trust. That we want you to get well, and we want to take care of your anxiety and your depression.” (Supervisor, mental health care)

However, care managers also used habitual strategies to develop service users’ trust in them which could challenge the ideal about knowledge-sharing during roundtable meetings. A care manager described that trust in the dyadic relationship between him and the service user was dependent on the promise of medical confidentiality:

“You need that trust in you to have a therapeutic alliance. Well, people must be certain that I will not discuss the things they disclosed to me with a third party. That simply would not be ethically sound. I could tell that we are working on some issues that she has with setting boundaries. But the details about it […], I would not say that out loud at the roundtable unless she mentioned it herself.” (Care manager)

#### Employment consultants’ strategies

Employment consultants applied different strategies to develop service users’ and care managers’ trust in them despite the potential conflict of interest between the jobcentre and the service users. This section describes two types of strategy that were sometimes described as positive and sometimes problematic. Some employment consultants showed trustworthiness by indicating that the service user’s interests were the most important to them, which was perceived positively by care managers and service users:

“It makes me feel safe in some way. That if I am not ready in a month then I don’t have to. Because the employment consultant said it out loud: ‘though there is this sickness benefit reform, that does not matter, because you will get better’. It is sort of a buffer in case I continue to talk nonsense.” (Service user, initial interview)

In some situations, this approach involved construing jobcentre interests as service-user interests in dialogue with service users:

“Through conversation, I just like to make the service users realise that they won’t get well by staying in this system. There is evidence that the faster you get back [to work], the… Sometimes I have to get tough, but then maybe it is also for their own sake, right?” (Employment consultant)

Another type of strategy involved creating transparency about the interests of the jobcentre and reliability regarding the complicated requirements of the legislation:

“We talk about the ‘reassessment time’ from the first session. I do not decide that the case is closing. It does that completely automatically. Before we get there, I need to find out if this person fits into one of seven boxes [legal paragraphs]. If I can’t fit them into any box, then they get transferred to the reassessment programme [lower benefit level]. And that is not personal or anything, that is simply how the law works.” (Employment consultant)

This type of strategy could be used to present trustworthiness by distancing herself from the legislation when she separated herself from ‘the system’.

However, the latter type of communication was sometimes disputed and perceived as threatening or unsympathetic by care managers or service users when presented during roundtable meetings. Likewise, the first type of strategy also sometimes caused a breach in trust (e.g. when employment consultants in rare cases had to impose decisions on service users) which was described as more uncomfortable than usual by an employment consultant.

#### Strategies supporting trust between professionals

Supervisors expressed in unity that interpersonal trust developed in the relationships between professionals from jobcentres and mental health care centres which compensated for the latent distrust between the involved organisations. Supervisors and professionals expressed that the personal acquaintance between professionals as well as care managers understanding of the employment consultants’ premises for work was crucial for developing trust:

“It is about the personal acquaintance. You know who the person is. You have an idea about their ‘basic assumptions’ in life. You know about their scope for action. […] It turns out that they [employment consultants] are friendly, co-operative and hard-working persons operating within the limits of their work.” (Supervisor, mental health care)

Professionals and supervisors in both organisations expressed that it was important that care managers felt the employment consultants genuinely wished to act in the interests of the service users. A supervisor explained why trust had evolved between professionals:

“Because the health care professionals now know we have the best intentions. We want to bring them [service users] to a better place than they are the day we meet them, where they will be able to provide for themselves and regain agency. That care managers actually believe this.” (Supervisor, Jobcentre)

Professionals and supervisors found that trust-based working relationships had also improved because care managers refrained from questioning legal decisions (and sometimes showed initiatives to support decisions).

“We are in this collaboration where we have decisional power. It is important that they trust us to make reasonable decisions.” (Supervisor, jobcentre)

## Discussion

Trust between stakeholders is described as important on all organisational levels in integrated services (including on a micro-level) [17] and for planning and facilitating good return-to-work processes [52]. This study corroborates these finding and provides insight into why interpersonal trust is perceived as particularly important on a micro-level and how interpersonal trust is sought to be nurtured in this specific context.

### Context and compensating interpersonal trust

The organisational context shaped both the need for trust and premises for professionals’ trust-building. This study showed varying degrees of scepticism and abstract distrust towards the jobcentre ‘system’, and an institutional distrust [12] between jobcentres and health care institutions, both notions are supported by previous Danish research [53,54,55,56,57]. Supported by the supposition that vulnerability and uncertainty enhance the need for trust between all three stakeholders [6,9,50], we also found that the jobcentre context enhanced the need for trust between stakeholders through shaping service users’ vulnerability in consultations with professionals and creating uncertainty about the employment consultants’ interests. However, the stakeholders’ perception of trust-building indicates that the interpersonal trust in the personal acquaintance between stakeholders appeared to compensate for these challenges shaped by the organisational context. Furthermore, some of the applied strategies directly addressed the challenges shaping the need for trust (e.g. caring for vulnerabilities and vocalizing the priority of service-user interests), indicating that trust-development might be supported by understanding and addressing the concrete causes of the need for trust.

The organizational context also shaped the logics and perspectives of care managers and employment consultants respectively; a caring logic inherent to the health care sector can conflict somewhat with the legislative and resource-focused logic inherent to the Jobcenters. Earlier, we showed that the professionals were enacting norms logics inherent to their host organizations that sometimes conflicted, but also that the negotiation and alignment of new shared norms in the intersectoral teams improved the collaboration [37]. Furthermore, the organisational context of the employment consultants provided difficult premises for trust-building strategies. However, the mental health care organisation was less visible and appeared to provide more favourable premises for the care managers’ trust-building strategies. Furthermore, therapists are often perceived as very trustworthy [58], and the care managers’ trustworthiness might be further supported by their role as therapists affiliated with the health care sector.

### Balancing interests and relations in integrated trust-dynamics

This study indicates that it was challenging for employment consultants to balance the legislative interests of the jobcentre and the interests of the service users in the integrated intervention. For instance, employment consultants’ strategies for developing interpersonal trust sometimes backfired when jobcentre interests were prioritised. The employment consultants’ dilemmas when balancing interests might have been difficult to articulate since the intervention and the evaluation were built on subtle presumptions that the interests of the service user and jobcentre were largely similar [22,23,39]. However, this study indicates that it might be relevant to refine the ways potential conflicts of interest between jobcentre and service users are acknowledged without being communicated as threats and thereby hindering interpersonal trust-building.

Moreover, although care managers’ unconditioned care was highly valued by service users, this study also indicates that the care managers’ attempt to nurture the relation between them and service users might compromise the trust-building with employment consultants: care managers who were limiting knowledge-sharing with reference to medical confidentially or questioning legal decisions might jeopardise their collaboration with employment consultants. Other Danish research has shown that health care professionals can act as advocates for persons on sick leave [57] or act as a ‘buffer’ towards the requirements of the jobcentre [59]. These dynamics also appeared sometimes in the IBBIS intervention. Therefore, considerations should be made as to how to balance the need for trust between professionals and service users with the need for trust between professionals.

### Power and trust

The employment consultants’ formal power over service users formed the need and premises for interpersonal trust in the integrated intervention. In the context of health- and social care, Grimen argues that trust and power are closely related and that professionals are particularly powerful when exercising discretions used to assess eligibility for benefits [60]. This dynamic might be particularly present when eligibility for benefits is based on non-objective disorders (such as stress), where health care professionals can provide medical ‘evidence’ for the benefit case [57]. However, neither the informal nor formal power relations between professionals and service users were addressed in manuals or protocols [36,61] or in the theory used to develop professional integration, relational coordination [62]. Therefore, we suggest that the issue of power might have been under-acknowledged and that the redistribution of power to care managers (through formal and informal discretions regarding service users’ illness and workability) might make the relation between service user and care manager particularly important.

### Strengths and limitations

This study is based on large empirical material, a rigorous analytical method, and a theoretically informed design. The study adds important knowledge about trust in integrated services by focusing on the stakeholders at the micro-level of integration whilst also acknowledging the influence from the organisational context. By elaborating on the widely used Rainbow model for integrated care and investigating one of the most prominent aspects of normative integration, trust, this study adds to one of the newly established core theories of integrated care and deepens the understanding of why normative integration is important and how it can be supported.

The trustworthiness (transferrability, credibility and dependability) of this study is assessed to be fair [51]. The setting shapes the transferability. The concrete responsibilities and regulations of the Danish jobcentres, the specific challenges of the target population, and the high level of social trust among Danes [63] challenges transferability. However, the mechanisms by which trust-based relationships can compensate for organisationally shaped distrust and uncertainty in asymmetrical power relations might be relevant in other integrated care settings where one professional has statutory power over the service user. Credibility was heightened by the large and varied multi-stakeholder sample, and the structured content analysis in stakeholder-differentiated tables, and the continuous discussions and reflections among the authors. Credibility was lowered due to coding by a single researcher (RMP) and the secondary nature of the analysis.

A considerable limitation of the study is the secondary analysis of merged empirical material [45] which also affects dependability negatively. All observations and 46 of 48 interviews were conducted to investigate the roundtable meeting, without a specific focus on trust. This implies a serious risk of ‘missing data’ [45]. However, the robustness of the findings is strongly supported by the stakeholders’ unprompted expressions and displays of (dis-) trust, vulnerability, and uncertainty in relation to the delivery of roundtable meetings and the intersectoral collaboration. Further, this limitation might have been reduced after conducting the two interviews with supervisors with a strict focus on the development of trust. Moreover, RMP, KHP, MM and LFE participated in the original process evaluation study of the 16 service users’ interviews conducted by KHP [43], which enhanced the basis for analytical rigour in the secondary analysis [47].

The recruitment of service users was done through professionals who might have refrained from inviting the most vulnerable individuals, either to protect the service user from the stressors of being observed in a sensitive situation or because the professionals preferred not to present difficult situations to the research team. Therefore, this study might not represent the most difficult cases. However, for ethical considerations, professionals were given the liberty to invite the researchers in cases where service users were mentally fit for observation and interview.

Most professionals were only interviewed once and only in connection with the roundtable meetings. Thus, their trust-building with other professionals was mostly described through their retrospective notions of trust-building with other professionals in general. The developmental perspective could have been further elucidated by follow-up interviews with each professional. However, the roundtable meeting represented a key setting for developing inter-professional trust, and consequently the meeting provided a suitable point of departure for having a rich dialogue on trust-building. The largest part of our empirical material describes the early phases of relation-building between service users and professionals in the first month of a programme that on average took six months. Only 6 service user interviews address the service users at the end of intervention (> six months). Therefore, the empirical material might not provide solid descriptions of later development phases and potential trust-breaches [6] from service users’ perspectives.

## Conclusion and implications

This study showed that interpersonal trust between service users, care managers and employment consultants compensated for a latent distrust between the mental health care centre and jobcentre, service users and care managers’ scepticism towards the Danish jobcentres, service users’ vulnerability due to a combination of their mental state and employment consultants’ power over them, and care managers’ and service users’ uncertainty about employment consultants. We identified strategies that often supported service users’ trust in professionals (time to establish personal relationships, care managers’ caring approach and a feeling of sharing interests with professionals) and trust between professionals (time to establish personal relationships and care managers’ acknowledgement of employment consultants’ premises for work). This study indicates that especially employment consultants faced challenges when developing service users’ trust whilst balancing the interests of the service user with those of the jobcentre.

We suggest that intervention developers and managers attempt to identify and address potential distrust, vulnerabilities and uncertainties inherent to future integrated interventions and their context. Vulnerabilities and uncertanties can rarely be eliminated completely but can be counterbalanced by a caring approach and the explicit intention to act in the service users’ interests. Intervention developers and managers should be particularly attentive to professionals who have formal or informal power over service users and are restrained from using these strategies whilst engaging in integrated interventions. Furthermore, it is important that professionals are supported to refine the ways conflicts of interest are acknowledged and de-tabooed without impeding interpersonal trust-building. Furthermore, it is recommended to structurally support the time-consuming process where micro-level stakeholders can get to know each other, understand each other’s perspectives and build trust between them.

## Additional File

The additional file for this article can be found as follows:

10.5334/ijic.5599.s1 Appendix 1. Example of stakeholder-differentiated content analysis inspired by Graneheim.

## Figures and Tables

**Table:** 

CM:	Care manager
EC:	Employment consultant
IBBIS:	Integreret Behandlings- og BeskæftigelsesIndsats til Sygedagpengemodtagere med depression, angst og stress [In Danish]
SU:	Service user
