# Supplementary material for: Developing Interpersonal Trust Between Service Users and Professionals in Integrated Services: Compensating for Latent Distrust, Vulnerabilities and Uncertainty Shaped by Organisational Context
Source: Int J Integr Care. 2021 Jul 1;21(3):1. doi: 10.5334/ijic.5599 (PMC8252972; doi:10.5334/ijic.5599)
Supplement: Appendix 1. — Example of stakeholder-differentiated content analysis inspired by Graneheim. [file ijic-21-3-5599-s1.pdf]

Appendix 1*: Example of stakeholder-differentiated content analysis inspired by Graneheim*

Stake-holder	Meaning unit	Latent condensed meaning unit	Stakeholder-specific theme	General theme
SU	“She [EC] said to me: “(distorted voice) Can you give me a single reason why I should not declare you fit for work?” That question does not open a dialogue with a vulnerable person! […] If IBBIS is supposed to have some sort of care-giving effect on a person who is vulnerable like I am, then you really need to change the rhetoric.” (SU 21, I2)	The SU describe herself as vulnerable and does not feel supported by the employment consultant who plans to determine the sickness benefit case	SUs are vulnerable due to the employment consultants’ statutory power over their sickness benefit case	SUs are vulnerable towards the ECs due to their statutory power over the sickness benefit case (power to promote return to work or determine sickness benefits)
CM	”They [SUs] are so vulnerable because they sit in front of a public official, an authority. Sometimes they get anxiety symptoms because they sit in front of this person [EC].” (CM1)	CM perceive the SUs to be vulnerable towards the employment consultant and fear this vulnerability worsens mental symptoms	CMs perceive SUs to be vulnerable due to the employment consultants’ statutory power over their sickness benefit case	
EC	”If you [SU] are sick and insecure and vulnerable, and you have experienced something at work, that was unhealthy for you, then naturally, you would be afraid to do it again. […] Because they [SUs] are very aware that the Jobcenter has some power over them. In that situation, they can start to worry, how are they [ECs] going to use that power?” (Supervisor 3)	Supervisor perceive the SUs to be insecure and vulnerable and relate this to the ECs power to accelerate their return to work plan or determine their sickness benefit	Supervisor perceives SUs to be vulnerable due to the employment consultants’ statutory power over their sickness benefit case	

EC: Employment consultant; CM: Care manager; SU: Service user
